# Parameter-Adaptive TVF-EMD Feature Extraction Method Based on Improved GOA

**DOI:** 10.3390/s22197195

**Published:** 2022-09-22

**Authors:** Chengjiang Zhou, Zenghui Xiong, Haicheng Bai, Ling Xing, Yunhua Jia, Xuyi Yuan

**Affiliations:** 1 School of Information Science and Technology, Yunnan Normal University, Kunming 650500, China; 2 The Laboratory of Pattern Recognition and Artificial Intelligence, Kunming 650500, China; 3 Faculty of Information Engineering and Automation, Kunming University of Science and Technology, Kunming 650500, China

**Keywords:** TVF-EMD, IGOA, *EEMI* index, bearing, signal decomposition

## Abstract

In order to separate the sub-signals and extract the feature frequency in the signal accurately, we proposed a parameter-adaptive time-varying filtering empirical mode decomposition (TVF-EMD) feature extraction method based on the improved grasshopper optimization algorithm (IGOA). The method not only improved the local optimal problem of GOA, but could also determine the bandwidth threshold and B-spline order of TVF-EMD adaptively. Firstly, a nonlinear decreasing strategy was introduced in this paper to adjust the decreasing coefficient of GOA dynamically. Then, energy entropy mutual information (*EEMI*) was introduced to comprehensively consider the energy distribution of the modes and the dependence between the modes and the original signal, and the *EEMI* was used as the objective function. In addition, TVF-EMD was optimized by IGOA and the optimal parameters matching the input signal were obtained. Finally, the feature frequency of the signal was extracted by analyzing the sensitive mode with larger kurtosis. The optimization experiments of 23 sets of benchmark functions showed that IGOA not only enhanced the balance between exploration and development, but also improved the global and local search ability and stability of the algorithm. The analysis of the simulation signal and bearing signal shows that the parameter-adaptive TVF-EMD method can separate the modes with specific physical meanings accurately. Compared with ensemble empirical mode decomposition (EEMD), variational mode decomposition (VMD), TVF-EMD with fixed parameters and GOA-TVF-EMD, the decomposition performance of the proposed method is better. The proposed method not only improved the under-decomposition, over-decomposition and modal aliasing problems of TVF-EMD, but could also accurately separate the frequency components of the signal and extract the included feature information, so it has practical significance in mechanical fault diagnosis.

## 1. Introduction

Signals contain rich feature information, so it is particularly important to extract key information through signal processing techniques [1]. In the early stage of bearing failure, the local surface damage will excite the transient impact of vibration signal. Therefore, finding damage-related transient shocks from complex vibration signals is the key to fault diagnosis [2]. However, the early fault diagnosis of bearings faces three great challenges: (1) the interaction between mechanical components and the environment causes the coupling between non-stationary vibration signals and noise signals [2]; (2) repeated transients induced by faults are hidden in noise and interference components [3]; and (3) compared with the natural frequency of bearing, the characteristics of slight damage fault are not obvious.

At present, two types of methods are commonly used to solve the above problems, including spectral analysis and envelope demodulation [4]. Spectral analysis locates the resonant frequency bands by establishing the spectral frequency and cyclic frequency planes, but most spectral analysis methods are time-consuming and memory-intensive. Envelope demodulation preserves the effective resonant frequency band by bandpass filtering and performs envelope analysis. However, finding the most suitable demodulation frequency band is the main challenge of this method [5]. At present, researchers determine the most appropriate demodulation frequency band through the following three methods: (1) detect the impulse sequence in the signal through bandpass filter and kurtosis. Popular methods include spectral kurtosis (SK), infographic, etc. [6]; (2) the demodulation frequency band is determined by detecting the periodic shocks of the signal, and the classical methods include minimum entropy deconvolution (MED), maximum correlation kurtosis deconvolution (MCKD), and so on [7]; and (3) signal decomposition is another effective method. Common decomposition methods include wavelet packet decomposition (WPD), local mean decomposition (LMD), empirical mode decomposition (EMD), etc. In addition, intrinsic time scale decomposition (ITD), variational modal decomposition (VMD) and empirical wavelet transform (EWT) are novel methods. However, these methods have some inherent defects. WPD will produce frequency aliasing and false frequencies [8]. EMD and LMD methods have the problems of modal aliasing and endpoint effect [9]. ITD adopts a linear transformation strategy, which leads to waveform blur and distortion [10]. The mode number and penalty parameters of VMD need to be set in advance, when the parameters are not suitable, over-decomposition or under-decomposition will occur [11]. The Fourier segmentation required by EWT strongly depends on the local maxima of the Fourier spectral amplitude, which means that the reliability of the Fourier segmentation is low [12].

Heng Li proposed time-varying filtering empirical mode decomposition (TVF-EMD) [13]. Compared with the above methods, this method has the following advantages [14]: (1) a time-varying filter is used in the transformation process of TVF-EMD, which can improve the mode aliasing while maintaining the time-varying characteristics of the algorithm; (2) the improved stopping criterion gives TVF-EMD robust performance at low sampling rate.; and (3) this method is insensitive to noise. However, we need to pre-set two key parameters of TVF-EMD. The bandwidth threshold affects the performance of modal separation, and the order of B-spline affects the filtering performance of the algorithm. When the parameters are selected appropriately, TVF-EMD also has two other advantages: (1) the screening process is completed by the optimal time-varying filter (B-spline approximate filter), which effectively solves the problem of mode aliasing in the decomposition process; and (2) the separation and intermittency problems in the decomposition process are completely solved. It can be seen that the parameter selection of TVF-EMD is particularly important. Inspired by the application of natural heuristic method in signal processing, this paper considers using a bionic algorithm to adaptively determine the parameters of TVF-EMD.

The application of optimization algorithm in signal processing is gradually increasing, which not only avoids the influence of human experience, but also improves the signal processing performance of the corresponding methods. The performance of signal decomposition largely depends on the segmentation effect of the intrinsic mode function (IMF). Those sub signals with the same zero crossing points and extreme points are called standard IMF, and several IMFs can form any signal. Wang et al. [15] proposed an improved VMD based on the Archimedes optimization algorithm (AOA) and used the AOA algorithm to find IMFs that were sensitive to fault features. Jin et al. [16] proposed an improved gray wolf optimization (GWO) algorithm based on hybrid strategy, and the improved GWO algorithm was used to optimize VMD parameters for signal decomposition. However, this method relies on manually extracted features. Meng et al. [17] proposed a fault diagnosis method based on the autoregressive moving average (ARMA) model and the multi-point optimal minimum entropy deconvolution adjustment (MOMEDA) algorithm, and the MOMEDA parameters were optimized by using the sparrow search algorithm (SSA). Ji et al. [18] proposed an OWT structural performance degradation evaluation method based on an optimized variational modal decomposition (VMD) algorithm, which can extract structural performance degradation characteristics from noise reduction data. Lu et al. [19] proposed an improved VMD adaptive signal denoising method, which used the optimized VMD to decompose the pipeline leakage signal and obtain multiple eigenmode functions. MGA et al. [20] proposed a framework based on the sailfish optimization algorithm (SFO) and Gini index (GI), which adaptively selects the optimal VMD parameters for each fault signal. However, the VMD method consumes more time than other competitive algorithms. Zhang et al. [21] proposed a parameter-adaptive VMD method based on the grasshopper optimization algorithm (GOA) to analyze the vibration signals of rotating machinery. Mehdi et al. [22] used the grasshopper optimization algorithm (GOA) to determine the clustering center, and used transformer operation data to evaluate and compare the performance under different conditions. Hu et al. [23] used nonlinear strategy to improve the attenuation coefficient of GOA, and introduced golden sine operator to update the individual position of GOA. However, the calculation time of this method model is long. Yahya et al. [24] proposed an improved grasshopper optimization algorithm (GOA) to improve the exploration and development ability of GOA by adjusting the new function of GOA main control parameters, but this method is too complex and inflexible. Ahmed A et al. [25] proposed an improved grasshopper optimization algorithm (GOA) based on opposed learning (OBL) strategy, called OBLGOA, to solve benchmark optimization functions and engineering problems, but this method is more time-consuming than GOA. Therefore, we proposed a simple and effective improvement strategy in this paper, which dynamically adjusts the decline coefficient of GOA through the nonlinear decline strategy.

In the above optimization problems, the selection of the objective function directly determines the optimal solution of the problem. Cor [26], mutual information (MI) [27], Kurt, energy coefficient (EC) [28], and energy entropy (*EE*) [29] are commonly used evaluation indexes in signal processing. Other indexes include entropy, sparsity metrics, smooth metrics, etc. [30]. MI can characterize the interdependence and matching degree between two signals. If only MI is used as the objective function, the information related to the original signal will be preserved, and the information related to the fault may be lost. *EE* can characterize the energy distribution of the signal in the frequency-domain, and the energy distribution of the frequency is closely related to the operating state. If only *EE* is used as the objective function, the information related to the original signal may be lost. Considering the shortcomings of both, the energy entropy mutual information (*EEMI*) composed of *EE* and *MI* is used as the objective function.

To sum up, we proposed a parameter-adaptive TVF-EMD feature extraction method based on IGOA in this paper. First, the GOA decrement coefficient is adjusted through a nonlinear decrement strategy, which can not only balance exploration and development, but also enhance global and local search capabilities. Then, *EEMI* is introduced to consider the energy distribution of the modal and the dependence between the modes and the original signal, and take it as the objective function. Then, TVF-EMD is optimized by IGOA and the optimal parameters matching the input signal are obtained. Finally, sensitive modes with large kurtosis are analyzed to extract the characteristic frequency of the signal. The effectiveness of the proposed method is demonstrated by analyzing the simulation signals and the bearing test bench signals.

The arrangement of this paper is as follows: The Section 2 briefly introduces the theory of TVF-EMD and GOA; in the Section 3, the proposed method is introduced in detail; in the Section 4, the effectiveness of the proposed method is verified by 23 benchmark functions, simulation signals and bearing signals; the Section 5 draws a conclusion.

## 2. Methodology and Theory

### 2.1. Empirical Mode Decomposition of Time-Varying Filtering

EMD is an effective adaptive decomposition method. However, mode aliasing is the main disadvantage of EMD, and it also has intermittent and separation problems. In addition, EMD is extremely sensitive to noise and low sampling rate. To solve these problems, Heng Li proposed TVF-EMD.

In EMD, a given signal x(t) is decomposed into a set of single-component signals (i.e., IMF) and residuals, i.e.,

(1) $$x(t)=\sum_{i=1}^{N}imf_{i}(t)+r(t)$$

where $imf_{i}(t)$ represents the *i*-th IMF obtained by the decomposition algorithm, and r(t) represents the residual term obtained by the signal decomposition. The screening process of EMD consists of two steps [31]: (1) estimating the “local mean”, and (2) extracting the local mean from the given signal until the resulting signal meets the IMF standard.

In TVF-EMD, IMF is replaced by local narrowband signal, because the characteristics of local narrowband signal are similar to IMF, and it can provide meaningful Hilbert spectrum. The two phases of TVF-EMD are summarized below [32].

(1) Local cut-off frequency rearrangement

Step 1: Find the place where the break occurs by setting a threshold on the rate of change over a certain time span. These breaks satisfy the conditions:

(2) $$\frac{\max(\phi_{bis}^{\prime }(u_{i}:u_{i+1}))-\min(\phi_{bis}^{\prime }(u_{i}:u_{i+1}))}{\min(\phi_{bis}^{\prime }(u_{i}:u_{i+1}))}>\rho$$

where $u_{i}$ is the maximum time consumption of x and $\phi_{bis}^{\prime }$ is the bisection frequency. The time consumption of $u_{i}$ is regarded as a break $e_{j}(j=1,2,\cdots )$.

If $\phi_{bis}^{\prime }(u_{i+1})-\phi_{bis}^{\prime }(u_{i})>0$, $e_{j}$ is on the rising edge of $\phi_{bis}^{\prime }(t)$. If $\phi_{bis}^{\prime }(u_{i+1})-\phi_{bis}^{\prime }(u_{i})<0$, $e_{j}$ is on the falling edge of $\phi_{bis}^{\prime }(t)$.

Step 2: If $e_{j}$ is on the rising edge,$\phi_{bis}^{\prime }(e_{j-1}:e_{j})$ is regarded as the floor, if $e_{j}$ is on the falling edge, $\phi_{bis}^{\prime }(e_{j}:e_{j+1})$ is regarded as the floor, and the rest of $\phi_{bis}^{\prime }$ is regarded as the peak.

Step 3: By interpolation between peaks, the resulting local cutoff frequency can be obtained. In the local cutoff frequency rearrangement stage, TVF-EMD solves the separation problem as well as the intermittent problem.

(2) Screening process based on time-varying filtering

Step 1: Estimate the local cutoff frequency from the original signal x.

Step 2: Filter the given signal by TVF to obtain the local mean. During this process, the bandwidth threshold ξ determines whether a given signal should be filtered or not, as well as the separation effect. The B-spline order n has nothing to do with the cutoff frequency estimation, it determines the attenuation and filtering effect of the TVF.

Step 3: Check whether the residual signal satisfies the following stopping criteria.

(3) $$\theta (t)=\frac{B_{Loughlin}(t)}{\varphi_{avg}(t)}$$

where $B_{Loughlin}(t)$ is the Loughlin transient bandwidth of the two component signals and $\varphi_{avg}(t)$ is the weighted average transient frequency of the single component. The detailed calculation process of TVF-EMD can refer to [32,33].

### 2.2. Grasshopper Optimization Algorithm

GOA is a heuristic algorithm that simulates the foraging behavior of grasshoppers [34]. It has two characteristics: (1) the adult worm moves fast and has a wide range, and it is mainly used to explore the search space; and (2) the nymph moves slowly and has a small step size, which is used to develop candidate solutions near the target. The mathematical model of GOA is as follows:

(4) $$X_{i}=S_{i}+G_{i}+A_{i}$$

(5) $$\{\begin{array}{l} S_{i}=\sum_{j=1,j\neq i}^{N}s(d_{ij})\hat{d_{ij}}\\ G_{i}=-g\hat{e_{g}}\\ A_{i}=u\hat{e_{w}} \end{array}$$

where $X_{i}$ represents the position of the *i*-th grasshopper, $S_{i}$ represents the social influence factor, $G_{i}$ represents the gravity of the *i*-th grasshopper, and $A_{i}$ represents the horizontal flow of wind. N is the number of grasshopper populations, $d_{ij}$ is the distance between the *i*-th grasshopper and the *j*-th grasshopper, i.e., $d_{ij}=|x_{j}-x_{i}|$. $\hat{d_{ij}}=(x_{j}-x_{i})/d_{ij}$ is a unit vector from the *i*-th grasshopper to the *j*-th grasshopper, and g is the gravitational constant, $\hat{e_{g}}$ is the unit vector in the direction of gravity, u is constant drift, $\hat{e_{w}}$ is the unit vector of the wind direction. s is a function that defines the strength of social ability, which can be adjusted by changing f and l, as shown in Equation (6).

(6) $$s(r)=fe^{\frac{-r}{l}}-e^{-r}$$

where f is the intensity of attraction and l is the length scale of attraction. From all these values we have chosen l=1.5 and f=0.5. Substitute $S_{i}$, $G_{i}$ and $A_{i}$ into Equation (4), which is extended to:

(7) $$X_{i}=\sum_{j=1,j\neq i}^{N}s(|x_{j}-x_{i}|)\frac{x_{j}-x_{i}}{d_{ij}}-g\hat{e_{g}}+u\hat{e_{w}}$$

where $x_{j}-x_{i}$ is the location difference between the *i*-th grasshopper and the *j*-th grasshopper, $d_{ij}$, $\hat{e_{g}}$ and $\hat{e_{w}}$ has been defined above.

In fact, the model applied to the population is in free space. Therefore, we do not consider gravity (no G component) and assume that the wind direction is always towards the optimized target $\hat{T_{d}}$. The improved update method is as follows:

(8) $$X_{i}^{d}=c\left(\sum_{j=1,j\neq i}^{N}c\frac{ub_{d}-lb_{d}}{2}s(|x_{j}^{d}-x_{i}^{d}|)\frac{x_{j}-x_{i}}{d_{ij}}\right)+\hat{T_{d}}$$

where ubd and lbd are the upper and lower bounds of the D-dimensional space, respectively. $\hat{T_{d}}$ is the current optimal solution of the D-dimensional objective function, in order to coordinate exploration and development, the decrement coefficient c decreases as the iteration increases.

(9) $$c=c_{\max}-l\frac{c_{\max}-c_{\min}}{L}$$

where l is the current iteration number, L is the maximum number of iterations, $c_{\max}$ and $c_{\min}$ are the maximum and minimum values of c, respectively. In this paper, $c_{\max}$ and $c_{\min}$ are taken as 1 and 0.00001, respectively.

## 3. Proposed Method

### 3.1. Improved Grasshopper Optimization Algorithm

In Equation (8), the decreasing coefficient c appears twice. As the number of iterations increases, the inner c reduces the attraction or repulsion between grasshoppers. It is used to explore a larger search space. Outer c balances the exploration and development of groups near the target [35]. It is used to reduce the movement of grasshoppers near the target.

In GOA, c decreases linearly, which means that the interaction force between grasshoppers decreases linearly, and the moving speed of grasshoppers near the target also decreases linearly. Therefore, there are two flaws in this strategy: (1) the inner c decreasing speed is too fast, which will cause the force between the grasshoppers to be very small, so the large-scale parameter space will be missed; and (2) if the outer c decreases too slowly, the grasshopper near the target will move too fast, so the global optimal solution will be lost.

**Figure 1:**
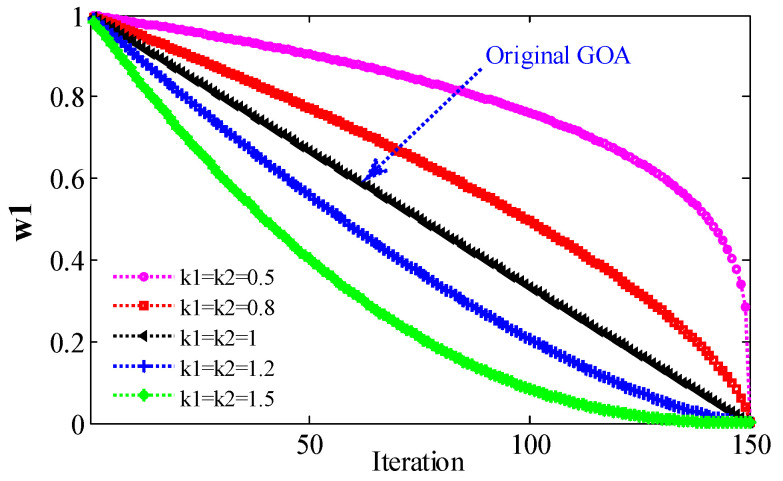
The relationship between ω1 and parameters k1, k2.

**Figure 2:**
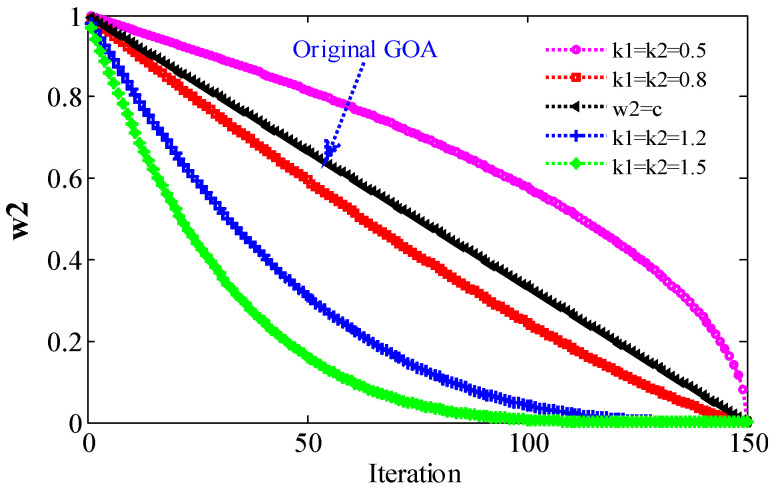
The relationship between ω2 and parameters k1, k2.

In order to solve the above problems, the nonlinear decline strategy is used to improve the decline coefficient c. Replace inner c with ω1 and outer c with ω2. The improved location update formula is shown below.

(10) $$\omega 1=c_{\min}+[(c_{\max}-c_{\min})*(1-\frac{l}{L}{)}^{k1}{]}^{k2}$$

(11) $$\omega 1^{\prime }=c_{\min}+(1-\frac{l}{L}{)}^{k1*k2}$$

(12) $$\omega 2={\left(\omega 1\right)}^{2}$$

(13) $$X_{i}^{d}{}^{\prime }=\omega 2\left(\sum_{j=1,j\neq i}^{N}\omega 1\frac{ub_{d}-lb_{d}}{2}s(|x_{j}^{d}-x_{i}^{d}|)\frac{x_{j}-x_{i}}{d_{ij}}\right)+\hat{T_{d}}$$

where k1 and k2 are natural numbers, and other parameters are the same as those of Formula (9). Since $c_{\min}=0.0001$, $c_{\max}=1$, ω1 is approximately equal to $\omega 1^{\prime }$, indicating that the same value of k1 and k2 has little effect on ω1. Therefore, in IGOA, the value of the ω1 strategy is k1=k2=0.8. At the same time, in order to compare the influence of different parameters on the results, k1 and k2 are set to 0.5, 0.8, 1, 1.2, and 1.5, respectively, in the following experiments. When the maximum number of iterations (Maxiter) L=150, the change trend of ω1 is shown in Figure 1. When k1=k2=1, Equation (10) is equivalent to (9), i.e., ω1 is the original linear decreasing strategy c. The change trend of ω2 is shown in Figure 2. When ω2=c, it is the decreasing strategy of the original GOA.

Compared with the original GOA (k1=k2=1), the decline rate of decline strategy ω1(inner c, k1=k2=0.8) is very slow. This means that the force between grasshoppers decreases slowly. Therefore, grasshoppers move rapidly in a wide parameter space and can explore a larger search space. From the theoretical analysis, the first problem of GOA can be solved.

Compared with the original GOA (k1=k2=1), the decrement strategy ω2 (outer c, k1=k2=0.8) has a fast decrement. This means that the movement speed of grasshoppers near the target decreases quickly. The grasshopper moves slowly in a small search space near the target, so the algorithm can obtain the global optimal solution. Theoretically, the second problem of GOA can be solved. The above two strategies are called IGOA, and the effects of different parameters will be discussed in the comparative experiments.

### 3.2. Construction of Optimization Model

In the stable operation stage, the signal energy depends on the bearing rotational frequency and its harmonics. When local damage occurs, the energy is gradually absorbed to the fault frequency [36]. Therefore, the failover information can be characterized by energy entropy (*EE*). In addition, the dependence between the original signal and the modes obtained by TVF-EMD can be characterized by mutual information (*MI*). Considering these two factors, we proposed the energy entropy mutual information (*EEMI*) index in this paper. In each decomposition process, the cumulative sum $\sum_{i=1}^{N}EEMI$ of the *EEMI* indexes of all IMFs is calculated. The maximization of $\sum_{i=1}^{N}EEMI$ is the optimization problem of this paper.

(14) $$\{\begin{array}{l} fitness=\underset{\gamma =\{\xi ,n\}}{\min}\{-\sum_{i=1}^{N}EEMI\}\\ \xi \in [0.1,0.8]\\ n\in [6,30] \end{array}$$

where fitness is the fitness and γ=(ξ,n) is the parameter to be optimized. In order to ensure the reliability of the parameter optimization, we optimize the two parameters within a relatively large parameter range. In order to ensure the reliability of the parameter optimization, we optimize the two parameters within a relatively large parameter range. The parameter range is ξ∈[0.1,0.8], n∈[6,30].

(15) EEMI=EE∗MI

For each IMF, *EEMI* is the product of *EE* and *MI*. The calculation of *EE* for each mode is as follows:

(16) $$E_{i}=\int_{-\infty }^{+\infty }|imf_{i}(t){|}^{2}dt,i=1,2,\cdots ,N$$

where $imf_{i}(t),(i=1,\cdots ,N)$ is the mode of different frequency bands, and $E=\{E_{1},E_{2},\cdots ,E_{N}\}$ is the energy distribution of the vibration signal in the frequency-domain. For ease of analysis, the energy is normalized, known as the energy coefficient (EC).

(17) $$\sigma i=Ei/E,(i=1,\cdots ,N)$$

where $E=E_{1}+\cdots +E_{N}$, the *EE* of the IMF is defined by the Shannon entropy.

(18) $$EE=-\sum_{i=1}^{N}\left(\sigma i)\ln(\sigma i\right)$$

*MI* can be used to measure the dependence between the original signal x(t) (denoted as X) and the mode $imf_{i}(t)$ (denoted as Y). *MI* is more efficient than Cor [27]. In the discrete domain, the mutual information between X and Y is defined as follows, which is equivalent to Equation (20).

(19) $$MI(X;Y)=\sum_{y\in Y}\sum_{x\in X}p(x,y)\log\frac{p(x,y)}{p(x)p(y)}$$

(20) MI(X;Y)=H(Y)−H(Y|X)

where p(x) and p(y) are the marginal probability distribution functions of X and Y, respectively, and p(x,y) represents the joint probability distribution function of X and Y. H(Y) is the marginal entropy of Y and H(Y|X) is the conditional entropy.

### 3.3. Proposed Parameter-Adaptive TVF-EMD

**Figure 3:**
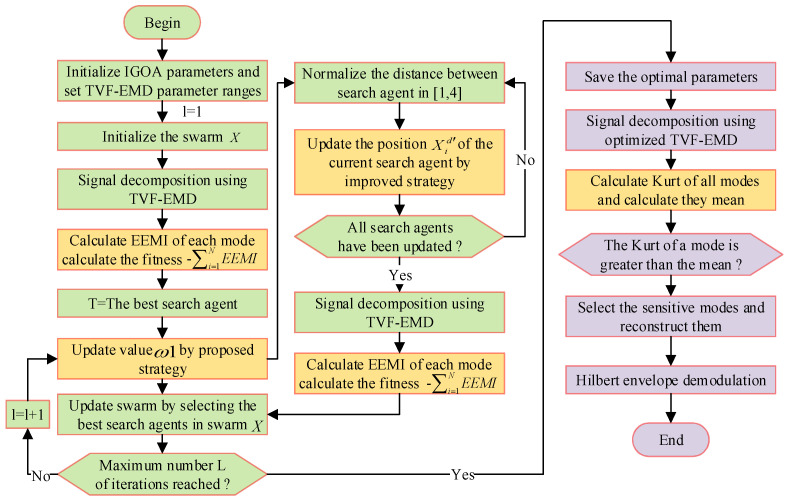
The calculation process of the IGOA-based TVF-EMD model.

As shown in Figure 3, the detailed steps of the proposed method are as follows: 

(1) Set the parameter range of TVF-EMD, and initialize the parameters of IGOA and population X, including search agent N, Maxiter *L*, k1 and k1.

(2) Use TVF-EMD to decompose the signal and calculate the *EEMI* index of the IMF to obtain the fitness $-\sum_{i=1}^{N}EEMI$. Store the best fitness and location.

(3) l=l+1, update the parameter ω1 using the improved strategy (10).

(4) Normalize the distance between search agents to be between [1, 4]. Update the location $X_{i}^{d}{}^{\prime }$ of the search agent using an improved strategy (13).

(5) Determine whether all search agents have been updated, and if not, execute (4). Otherwise, execute (6).

(6) Use TVF-EMD to decompose the signal and calculate the fitness $-\sum_{i=1}^{N}EEMI$, store the best fitness and position.

(7) Update the population *X* by obtaining the best search agent.

(8) Judge whether the Maxiter is reached, if not, execute (3)~(7). Otherwise, obtain the best fitness and parameter combination.

(9) Use the optimized TVF-EMD to decompose the signal, and use the IMFs with kurtosis larger than the mean kurtosis as the sensitive mode.

(10) Analyze the sensitive modes by Hilbert envelope demodulation.

## 4. Experimental Study

### 4.1. Function Optimization Experiment

To evaluate the optimization performance of IGOA, we optimize 23 benchmark functions [37], and the function information is shown in Table 1. All experiments are performed by MATLAB R2014a software under the environment of Intel(R) Core(TM) i5-5200U CPU @ 2.20 GHz 2.19 GHz, 8 g. To avoid random errors, we perform each method 30 times for each function. Meanwhile, the mean value (Avg), the best value (*Best*), the worst value (*Worst*), the standard deviation (*STD*), the success rate (*SR*), and the average time (Time) are used to evaluate the optimization results [24].

(21) $$Avg=\frac{1}{Nr}\sum_{i=1}^{Nr}F_{i}^{*}$$

(22) $$Best=\underset{1\leq i\leq Nr}{\min}(Fi^{*})$$

(23) $$Worst=\underset{1\leq i\leq Nr}{\max}(Fi^{*})$$

(24) $$STD_{F}=\sqrt{\frac{1}{Nr-1}\sum_{i=1}^{Nr}{\left(F_{i}-\mu F\right)}^{2}}$$

(25) $$SR=(\frac{\mathrm{Number}\;\mathrm{of}\;\mathrm{times}\;\mathrm{reached}\;\mathrm{VTR}}{Nr})$$

where Nr is the number of runs and $F_{i}^{*}$ is the fitness. Avg refers to the average fitness value in 30 runs, the smaller the average fitness, the greater the probability that the optimization algorithm will tend to the global optimization, and the better the result. $STD_{F}$ represents the discrete degree of the best fitness. The smaller the dispersion, the less the fitness deviates from the minimum value, and the better the stability of the optimization algorithm. SR refers to the ratio of times to reach the required value (VTR = 10E−5). The larger the A, the more times the optimization algorithm can reach the global optimal goal, and the better the reliability of the algorithm. Time refers to the average time of an algorithm at different runtimes.

#### 4.1.1. The Effects of Coefficients k1 and k2

Twenty-three kinds of benchmark functions are optimized through IGOA with different parameters, where the Maxiter is 50 and population size is 50. The parameters (k1, k2) of IGOA are 0.5, 0.8, 1, 1.2, 1.5, respectively. The results are shown in Table 2 and Table 3.

When k1=k2=0.5, except for F3, F5, F8, F14~F21, the Avg obtained by IGOA is larger than that obtained by GOA. Except for F1, F5, F8, F10, F14, F15, F17, F19, F21, the *STD* obtained by IGOA is larger than that obtained by GOA. The *SR* obtained by IGOA is smaller than that obtained by GOA. Therefore, when k1=k2=0.5, the optimization performance of IGOA is inferior to GOA, because the decreasing speed of ω2 is small (Figure 2). Because the grasshopper moves too fast near the target (problem 2 of GOA), the global optimal solution is lost. When k1=k2=0.8, except for F6, the Avg obtained by IGOA is smaller than that obtained by GOA. Except for F6, F18, and F20, the *STD* obtained by IGOA is smaller than that obtained by GOA. Except for F6, the *SR* obtained by IGOA is larger than that obtained by GOA. The results show that: (1) when k1=k2=0.8, compared with GOA, IGOA has stronger global and local search ability, higher stability and reliability; and (2) two problems of GOA have been improved.

When k1=k2=1.2 or k1=k2=1.5, the Avg obtained by IGOA is smaller than that obtained by GOA, except for F17, F22 and F23. Except for F9, F17, F19, F20, F22 and F23, the *STD* obtained by IGOA is smaller than that obtained by GOA. The *SR* obtained by IGOA is larger than that obtained by GOA. IGOA is better than GOA because ω2 decreases faster (Figure 2). Because the grasshopper moves slowly in a small search space near the target, the optimal solution can be obtained. However, compared with GOA, the optimization effect of these two strategies for functions with small parameter space is poor, and the stability is not high. This is because the reduction speed of ω1 is too fast, and the effective search space is omitted. The results show that: (1) when k1 and k2 are 0.8, 1.2, and 1.5, the optimization performance of IGOA is better than that of GOA, and the time consumed by IGOA and GOA is almost equal; (2) satisfactory optimized performance is obtained when the parameter of IGOA is 0.8; and (3) the ω1 and ω2 strategies are effective, both of which not only balance exploration and development, but also improve the global and local search capabilities of the original GOA. It shows that the proposed improvement strategy is effective and feasible.

#### 4.1.2. The Effects of the Maxiter and Population Size

The effects of the Maxiter and population size on optimization performance is analyzed through 15 benchmark functions. k1 and k2 are set to 0.8, and the population size is 50. The Maxiter is 100, 150, 200 and 300, respectively. The results are shown in Table 3 and Table 4 (Maxiter = 150).

When the Maxiter is 100, the Avg obtained by IGOA is smaller than that obtained by GOA except for F6 and F11, and the average time of both is about 11 s. When the Maxiter is 150 (Table 3), IGOA has been shown to be superior to GOA. When the Maxiter is 200, except for F1 and F11, the Avg obtained by IGOA is smaller than the Avg obtained by GOA, and the average time of both is about 21 s. When the Maxiter is 300, except for F1, F4, F6, and F11, the Avg obtained by IGOA is smaller than the Avg obtained by GOA, and the average time of both is about 32 s. The results show that: (1) with the increase in the Maxiter, Avg decreases, *SR* increases, and the optimization performance is enhanced. However, the time consumption increases; (2) for different Maxiter, IGOA is better than GOA; and (3) the Maxiter is not the bigger the better, and IGOA can achieve satisfactory results when the Maxiter is smaller. As a consequence, the practical problem can be solved by IGOA with a smaller Maxiter.

To explore the effect of population size on performance, k1 and k2 are set to 0.8, the Maxiter is 150, and population sizes are 30, 50, 100 and 200, respectively. The results are shown in Table 3 and Table 5. When the population size is 30, the Avg obtained by IGOA is smaller than that obtained by GOA except for F5 and F6, and the average time of both is about 6 s. When the population size is 50 (Table 3), IGOA achieves the best optimization performance compared with GOA. When the population size is 100, the Avg obtained by IGOA is smaller than that obtained by GOA except for F4, and the average time is about 61 s. When the population size is 200, except for F4 and F5, the Avg obtained by IGOA is smaller than that obtained by GOA, and the average time is about 253 s. The results show that: (1) as the population size increases, the Avg obtained for most functions decreases, but the *SR* also decreases and the time consumption increases; (2) for different population sizes, IGOA is better than GOA; (3) when the population size is 30~50, the optimization effect is better and less time-consuming; and (4) the population size is not the bigger the better. Compared with GOA, IGOA with smaller population size can achieve better optimization results.

#### 4.1.3. Comparison between IGOA and Other Methods

**Figure 4:**
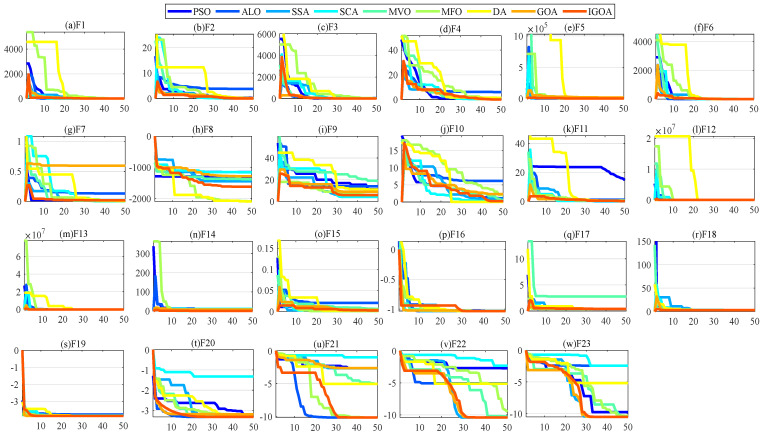
Convergence curves for all algorithms.

To demonstrate the superiority of IGOA, IGOA is compared with various optimization algorithms. As shown in Figure 4, these algorithms include particle swarm optimization (PSO), ant lion optimizer (ALO) [38], salp swarm algorithm (SSA) [19], sine cosine algorithm (SCA) [39], multi-verse optimizer (MVO) [40], moth–flame optimization algorithm (MFO) [41], dragonfly algorithm (DA) [42]. In order to ensure the fairness of the experiment, the Maxiter is set to 50, the population size is 20, and the parameters of all algorithms are default values. The results show that: (1) the convergence speed of IGOA (k1=k2=0.8) is very fast, and IGOA achieves the best optimization effect in the optimization of unimodal functions (F1~F7); (2) for multimodal functions (F8~F23), compared with other algorithms, the fitness obtained by IGOA is very small except for F8, F9; (3) compared with other algorithms, IGOA has fast convergence speed and strong global and local search ability; and (4) in F7, F8, F9, F10, and F21, the best fitness obtained by IGOA is smaller than that obtained by GOA. It is shown that IGOA improves the local optimal problem and slow convergence of GOA, and the effectiveness of the improved strategies ω1 and ω2 is proven.

### 4.2. Analysis of Simulated Signals

#### 4.2.1. Simulation and Comparison

**Figure 5:**
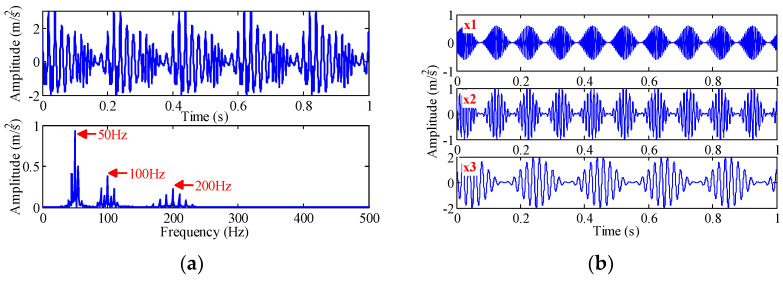
(**a**) Simulated signal x(t) and its frequency-domain waveform, and (**b**) its three components.

We analyzed several sets of amplitude-modulated and frequency-modulated (AM-FM) signal x(t) and the noisy linear signal y(t) by the proposed method. The signal x(t) is shown below, and the sampling frequency is 3000 Hz. The time-domain and frequency-domain waveforms and component signals of the signal are shown in Figure 5. The dominant frequencies of the components are 50 Hz, 100 Hz and 200 Hz, respectively.

(26) $$\{\begin{array}{l} x(t)=x_{2}(t)+x_{2}(t)+x_{3}(t)\\ x_{1}(t)=(0.3+0.3\sin(20\pi t))\times \cos(400\pi t+1.5\sin(20\pi t))\\ x_{2}(t)=(0.5+0.5\sin(20\pi t))\times \cos(200\pi t+0.5\sin(10\pi t))\\ x_{3}(t)=(1+\sin(10\pi t))\times \cos(100\pi t+0.3\sin(10\pi t)) \end{array}$$

**Figure 6:**
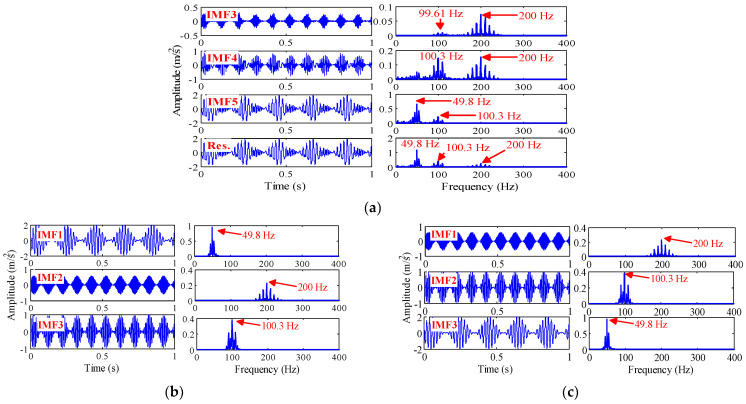
The decomposition results of simulated signal x(t) obtained by: (**a**) EEMD; (**b**) VMD; and (**c**) TVF-EMD.

The signal is decomposed by EEMD, VMD and TVF-EMD. The noise amplitude of EEMD is set to 0.3 and the integration number is 500 [18]. Since the signal x(t) contains three components, the number of modes of the VMD is three and the penalty factor is 2000. The bandwidth threshold and B-spline order of TVF-EMD are 0.1 and 20, respectively. The results are shown in Figure 6.

By the EEMD method, the signal x(t) is decomposed into 12 IMF. Figure 6a provides IMF3, IMF4, IMF5, and Res, which are closest to the simulated components, where Res is the reconstructed signal composed of the remaining nine components (except IMF3, IMF4, IMF5). As shown in Figure 6a, the frequency of 200 Hz appears in IMF3, IMF4 and Res. The frequencies of 100.3 Hz and 49.8 Hz appear in IMF5 and Res. A mode contains multiple dominant frequencies, and mode aliasing and frequency aliasing are obvious. As shown in Figure 6b,c, the decomposition results obtained by VMD and TVF-EMD are close to the simulated components, and aliasing phenomenon is not found. It is shown that the decomposition performance of VMD and TVF-EMD is better than that of EEMD in multi-component AM-FM signal decomposition.

We use three methods to decompose the noisy linear signal y(t). It consists of white noise with standard deviation of 0.1 and three linear signals.

(27) $$\{\begin{array}{l} y(t)=y_{2}(t)+y_{2}(t)+y_{3}(t)+n(t)\\ y_{1}(t)=1.8\times \cos(75\times 2\pi t)\\ y_{2}(t)=1.2\times \cos(25\times 2\pi t)\\ y_{3}(t)=0.5\times \cos(15\times 2\pi t) \end{array}$$

**Figure 7:**
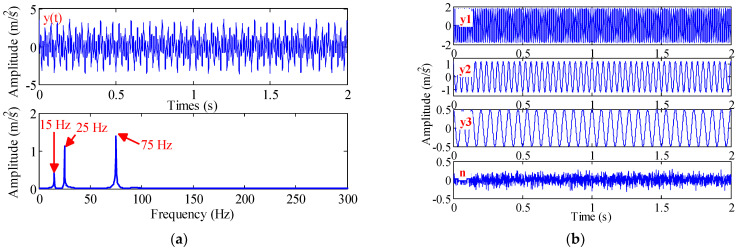
(**a**) Simulated signal y(t) and its frequency-domain waveform, and (**b**) its three components and noise signal.

**Figure 8:**
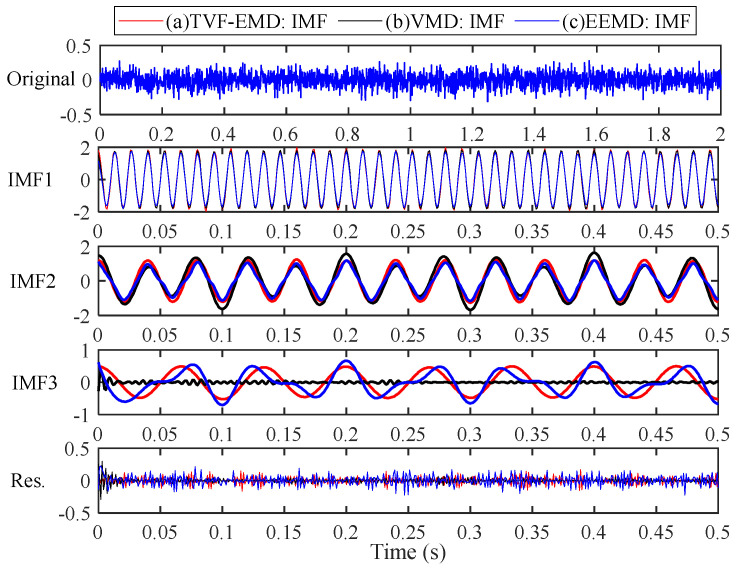
The decomposition results of simulated signal y(t) obtained by: (**a**) red line: TVF-EMD; (**b**) black line: VMD; (**c**) blue line: EEMD.

The time-domain and frequency-domain waveforms and component signals of y(t) are shown in Figure 7, where the frequencies of $y_{2}(t)$ and $y_{3}(t)$ are very close, and the sampling frequency is 1000 Hz. The noise amplitude of the EEMD is 0.3 and the number of integration is 500. Since y(t) is composed of four components, the mode number of VMD is set to 4 and the penalty factor is 2000. The bandwidth threshold and B-spline order of TVF-EMD are set to 0.1 and 26, respectively. The results are shown in Figure 8.

Eleven IMFs can be obtained by the EEMD method and four IMFs can be obtained by the TVF-EMD method. To facilitate comparison, the component signal of 0.5 s is used for analysis, and the corresponding results are shown in Figure 8. The IMF1, IMF2 obtained by EEMD and TVF-EMD are very close to the simulated components $y_{1}(t)$ and $y_{2}(t)$. However, the waveform (blue) of IMF3 obtained by EEMD is distorted, indicating mode aliasing has occurred in EEMD. The amplitude of IMF2 obtained by VMD is irregular, and noise is mixed in IMF3, and $y_{3}(t)$ is lost. TVF-EMD achieved satisfactory decomposition results.

The experimental results show that: (1) for the signals x(t) and y(t), the EEMD method has mode aliasing and frequency aliasing; (2) VMD can decompose the AM-FM signal x(t) effectively. However, mode aliasing occurs in the VMD in the decomposition of the noisy linear signal y(t), indicating that the VMD is sensitive to noise; (3) for the signals x(t) and y(t), the TVF-EMD method can achieve satisfactory decomposition results; and (4) the parameter selection has a great influence on the decomposition result. The effect of parameter selection on the decomposition performance of TVF-EMD will be studied in the following.

#### 4.2.2. The Effects of TVF-EMD Parameters

**Figure 9:**
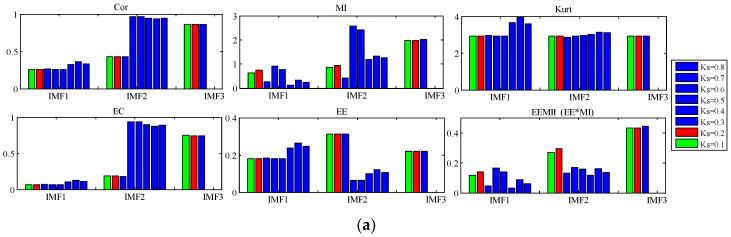

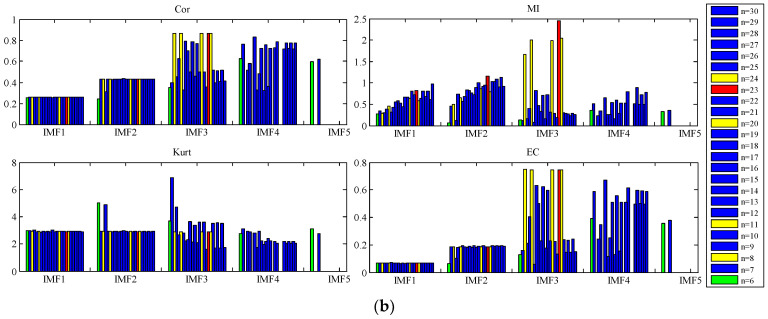
The relationship between the indexes of the IMF and the parameters (**a**) bandwidth threshold ξ, and (**b**) B-spline order n for simulated signal x(t).

**Figure 10:**
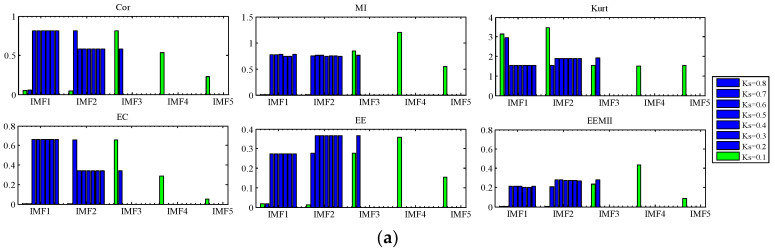

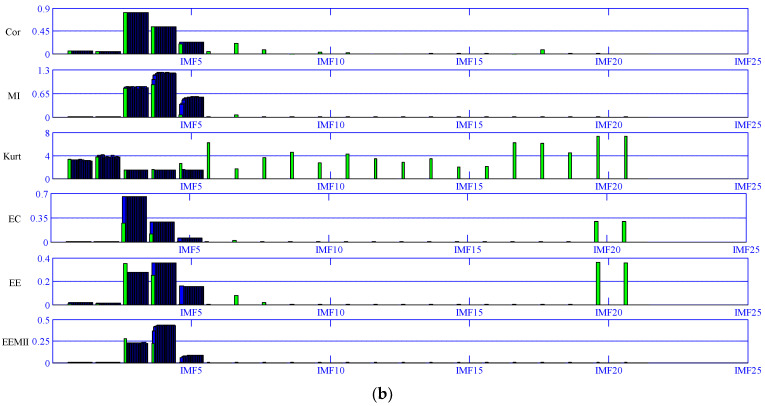
The relationship between the indexes of the IMF and the parameters (**a**) bandwidth threshold ξ, and (**b**) B-spline order n for simulated signal y(t).

1. Analysis of the simulated signal x(t)
  In the decomposition of AM-FM signal x(t), we study the effect of bandwidth threshold ξ and B-spline order n on the decomposition result of TVF-EMD. n is set to 20 and ξ is increased from 0.1 to 0.8. As shown in Figure 9a, we analyze the resulting IMFs by Cor, MI, Kurt, EC, *EE* and *EEMI* (green indicates the starting value of the parameter, and red indicates the best result). When ξ∈[0.4,0.8], two IMFs are obtained by decomposition. Mode aliasing and under-decomposition occur, and the three components of x(t) cannot be separated. When ξ∈[0.1,0.3], three IMFs are obtained by decomposition. When ξ increases from 0.1 to 0.3, the Cor, Kurt, EC, and *EE* indexes of IMF1, IMF2, and IMF3 do not change much, indicating that these indexes are not sensitive to the change of ξ. MI and *EEMI* are sensitive to ξ, but their changes are irregular when ξ increases from 0.1 to 0.3. When ξ=0.2, the sum of the *EEMI* indexes of IMF1, IMF2, and IMF3 is the largest, so it can be used as the selection rule for the best parameters. The results show that: (1) under-decomposition and mode aliasing will occur when ξ is too large; (2) MI and *EEMI* are sensitive to ξ, while other indexes are not sensitive to ξ; (3) the effects of ξ on the decomposition results are not regular; and (4) the introduced *EEMI* index and optimization model $-\sum_{i=1}^{N}EEMI$ are effective.
  Next, the bandwidth threshold ξ is set to 0.1, and the B-spline order n is increased from 6 to 30. The results are shown in Figure 9b. When n=6 or n=9, five IMFs are obtained by decomposition. When n takes values other than 6, 8, 9, 11, 20, and 24 in [6, 30], four IMFs are obtained by decomposition. In these two values, over resolution and modal aliasing occur. When n is 8, 11, 20, 23, 24 (yellow), tree IMFs are obtained by decomposition. When n varies in 8, 11, 20, 23, and 24, the Cor, Kurt, EC, and *EE* indexes of IMF1, IMF2, and IMF3 do not change much, indicating that these indexes are not sensitive to n. *MI* and *EEMI* are sensitive to n, but their changes are irregular. In addition, when n=23, the sum of *EEMI* of IMF1, IMF2, IMF3 is the largest, indicating that the optimization model based on *EEMI* is effective. The results show that: (1) over-decomposition and modal aliasing will occur when n is not selected properly; (2) MI and *EEMI* are sensitive to n, and other indexes are not sensitive to n; (3) the effects of n on the decomposition results are not regular; and (4) the introduced *EEMI* index and optimization model are effective.
2. Analysis of the simulated signal y(t)
  In the decomposition of the noisy linear signal y(t), we analyze the influence of two parameters on the decomposition results. The B-spline order n is set to 26, and the bandwidth threshold ξ increases from 0.1 to 0.8, as shown in Figure 10a. When ξ is 0.1, five IMFs are obtained by decomposition. When ξ is 0.2, three IMFs are obtained by decomposition. When ξ∈[0.3,0.8], two IMFs are obtained by decomposition. Because y(t) contains four components, under-decomposition and mode aliasing occur when ξ∈[0.2,0.8]. When ξ changes from 0.3 to 0.8, the Cor, Kurt, EC, and *EE* indexes of IMF1 and IMF2 change little, indicating that these indexes are not sensitive to ξ, while MI and *EEMI* are sensitive to ξ. The experimental results show that: (1) when ξ is too large, under-decomposition and mode aliasing will occur; and (2) MI and *EEMI* are sensitive to ξ, and other indexes are not sensitive to ξ.

Next, the bandwidth threshold ξ is set to 0.1, and the B-spline order n is increased from 6 to 30, as shown in Figure 10b. When n=6, 21 IMFs are obtained by decomposition, indicating that n is not selected properly and over-decomposition occurs. When n∈[7,30], five IMFs are obtained. When n=6, the Kurt, EC, and *EE* indexes of IMF19 and IMF21 are relatively large. If these indexes are used as the objective function, the parameters obtained will lead to over-decomposition. When n=6, the Cor and MI of multiple IMFs are also larger, and the *EEMI* indexes are mainly concentrated in IMF3, IMF4, and IMF5, indicating that these three modes are effective modes. The results show that: (1) over-decomposition and mode aliasing will occur when n is not selected properly; and (2) the *EEMI* index mainly focuses on the effective mode and is sensitive to n, so *EEMI* is effective.

#### 4.2.3. Validation of the Proposed Method

The above results show that the effects of ξ and n on the results are cross-coupled, and the effects of the two parameters on the results are irregular, and it is particularly difficult to determine the appropriate parameter group. Therefore, we determine the optimal parameters of TVF-EMD by GOA and IGOA, and analyze the signals x(t) and y(t) with this method. The Maxiter L is 10, the population size N is 30, and k1 and k2 are 0.8.

**Figure 11:**
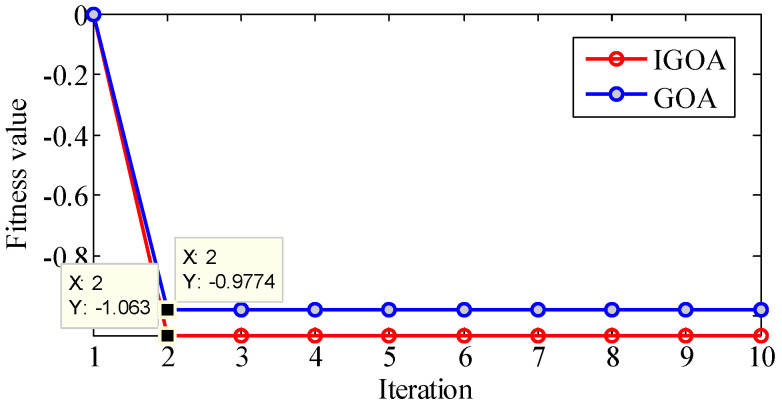
The optimization of TVF-EMD in the decomposition of simulation signal x(t).

For signal x(t), the optimization results are shown in Figure 11. The best fitness obtained by GOA is −0.9774, and the best parameters are ξ=0.174669, n=23; the best fitness obtained by IGOA is −1.063, and the best parameters are ξ=0.145251, n=23. Compared with GOA, the convergence accuracy of IGOA is higher. Since the waveforms obtained by TVF-EMD after optimization are very similar, the decomposition results are evaluated by Cor, MI, Kurt, EC, *EE*, *EEMI*, center frequency (CF) [28], root mean square error (RMSE) and energy leakage ratio (ELR) [38].

By specifying the parameters (ξ=0.1,n=20) in TVF-EMD, x(t) is decomposed into four IMFs, and the over-decomposition phenomenon occurs. The results are shown in Table 6. Compared with other modes, the evaluation indexes of IMF3 are very small, and the CF (54.93 Hz) of IMF3 does not match the frequency of the x(t) component, indicating that IMF3 is a false mode. The RMSE and ELR indexes are both larger than those obtained by the optimized TVF-EMD, indicating that inappropriate parameters will lead to larger decomposition errors and energy leakage.

Through the optimization of GOA and IGOA, x(t) is decomposed into three IMFs, and the over-decomposition and under-decomposition problems are solved. The Cor, MI, Kurt, EC, *EE*, and *EEMI* indexes of IMF1, IMF2, and IMF3 obtained by IGOA are all larger than those obtained by GOA, indicating that the mode obtained by decomposition are closer to the original components, and the decomposition performance is improved. The RMSE and ELR obtained by IGOA are smaller than those obtained by GOA, indicating that the decomposition error and energy leakage are reduced by the proposed method.

**Figure 12:**
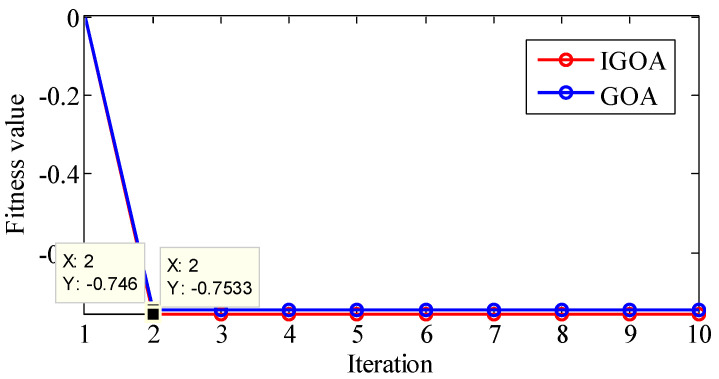
The optimization of TVF-EMD in the decomposition of simulation signal y(t).

For signal y(t), the optimization results are shown in Figure 12. The best fitness obtained by GOA is −0.746, and the best parameters are ξ=0.117211, n=17; the best fitness obtained by IGOA is −0.7533, and the best parameters are ξ=0.107442, n=27. It shows that the convergence accuracy of IGOA is higher than that of GOA.

Through the optimization of GOA and IGOA, y(t) is decomposed into five IMFs. The results are shown in Table 7. The Cor, MI, *EE*, and *EEMI* indexes of each mode obtained by IGOA are all larger than those obtained by GOA, indicating that the correlation between the obtained mode and the simulated components is improved. Except for Kurt, the indexes of IMF1 and IMF2 are all smaller than those of IMF3, IMF4, and IMF5, indicating that the high-frequency noise n(t) is decomposed into IMF1 and IMF2. The RMSE and ELR obtained by IGOA are smaller than those obtained by GOA, indicating that the decomposition error and energy leakage are reduced by the proposed method. The results show that: (1) compared with GOA, the proposed IGOA can achieve better optimization performance, and the improvement strategy of IGOA is effective; (2) the proposed optimization model is effective, and the *EEMI* index is not only sensitive to parameters but can also characterize the signal information more comprehensively; and (3) through the proposed method, the problems of parameter selection, under-decomposition, over-decomposition and mode aliasing that exist in the TVF-EMD method are resolved.

### 4.3. Signal Analysis of CWRU Rolling Bearing

**Figure 13:**
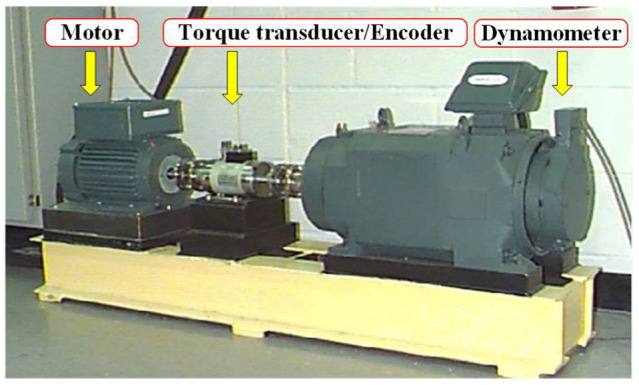
Bearing test rig.

The proposed method is used to analyze the bearing signal of Case Western Reserve University (CWRU). The experimental platform [43] consists of a 2 HP Reliance motor (left), a torque sensor (middle), a dynamometer (right) and control electronics (not shown), as shown in Figure 13. The motor speed is 1797 rpm (the rotational frequency Fr is 1797/60 Hz = 29.95 Hz), and the bearing parameters are shown in Table 8. In order to simulate the early failure or mild damage of the inner ring, outer ring and rolling element, the vibration signal with a small damage diameter (0.007 inches, about 0.01778 cm) is used for analysis. The sampling frequency is 12 KHz, and the experimental data length is 2048. According to the formula in [44] and the parameters in Table 8, the fault feature frequency of the bearing is obtained, as shown in Table 9.

#### 4.3.1. Analysis of Outer Ring Vibration Signal

According to the frequency-domain waveform of the inner ring signal, the characteristic frequency Fi can be clearly identified, so there is no need to further analyze the inner ring signal.

**Figure 14:**
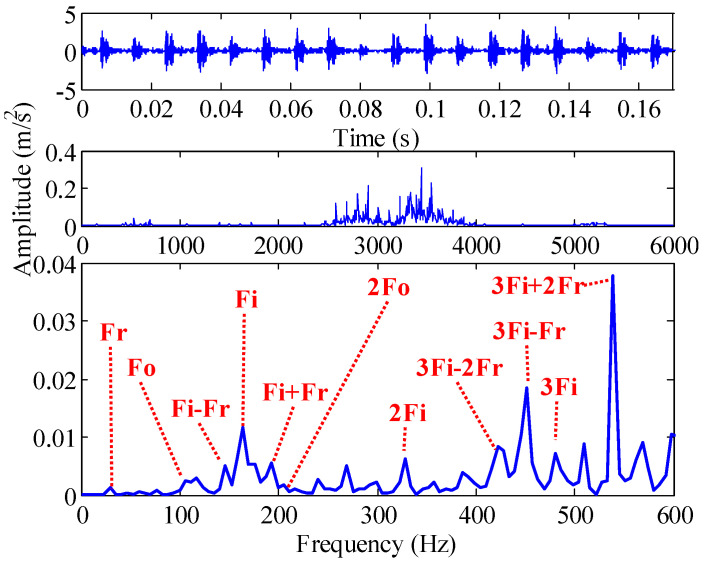
Time-domain and frequency-domain waveform of outer ring slight damage signal.

The time-domain and frequency-domain waveforms of the outer ring signal are shown in Figure 14. The outer ring frequency Fo is submerged by the interference frequency, so we analyze the outer ring refinement spectrum in the range of [0 Hz, 600 Hz]. The inner ring frequency Fi, modulation frequency 3Fi – Fr, and 3Fi + 2Fr have the largest spectral peaks. In addition, the modulation frequencies between Fi, 2Fi, 3Fi and rotational frequency can be clearly observed. According to the spectrum, the bearing condition is misdiagnosed as inner ring fault. In fact, the outer ring of this bearing suffered minor damage. However, the amplitude of the outer ring frequency Fo is very small compared with other frequencies, and it is almost submerged in the interference frequency.

**Figure 15:**
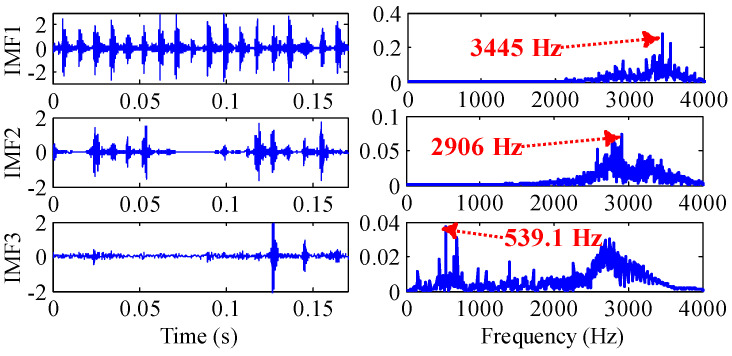
The IMFs obtained by TVF-EMD with assigned parameters.

The outer ring signal is decomposed by specifying parameters (ξ=0.2, n=6) TVF-EMD, as shown in Figure 15. The high-frequency components IMF1 and IMF2 are obtained by decomposition, but the low-frequency components contained in IMF3 are not successfully decomposed. In addition, the frequencies of IMFs obtained by decomposition are aliased with each other, which indicate that the parameter setting is inappropriate.

**Figure 16:**
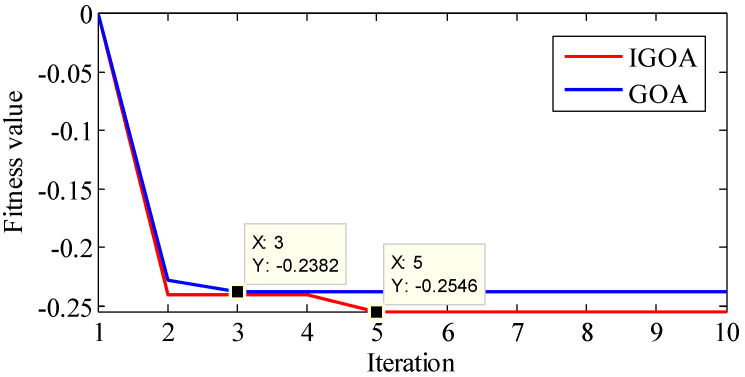
The fitness curve obtained by IGOA and GOA during the decomposition of the outer ring signal.

The signal is analyzed by the proposed method. First, TVF-EMD is optimized by IGOA and GOA, and the convergence curve is shown in Figure 16. The best fitness obtained by GOA is −0.2382, and the best parameters are ξ=0.28026, n=7. The best fitness obtained by IGOA is −0.2546, and the best parameters are ξ=0.238489, n=15. It shows that IGOA has higher convergence accuracy.

**Figure 17:**
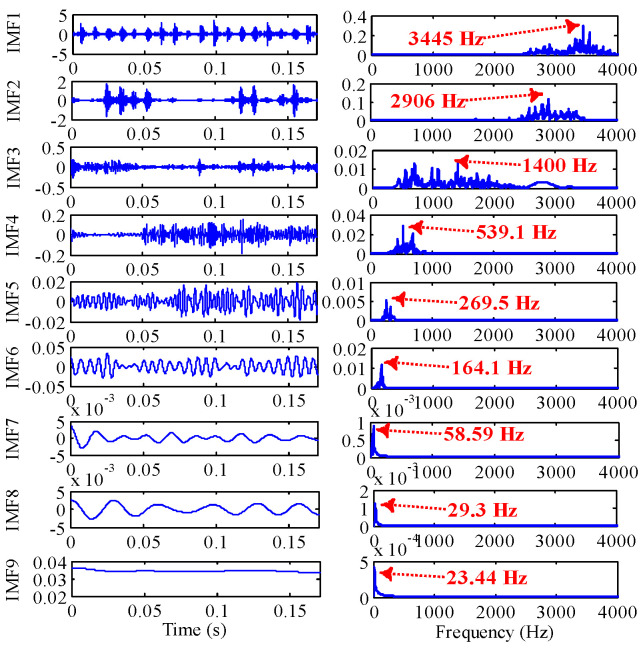
The IMFs of outer ring obtained by the optimized TVF-EMD method based on IGOA.

As shown in Figure 17, through the TVF-EMD method optimized by IGOA, the signal is decomposed into nine IMFs. IMF1, IMF2 and IMF3 are high-frequency noises, the center frequency of IMF4 (539.1 Hz) is close to 5Fo (5Fo = 536.8 Hz), the center frequency of IMF5 (269.5 Hz) is close to 2Fo (2Fo = 282.32), the center frequency of IMF6 (164.1 Hz) is close to Fi, the center frequency of IMF7 (58.59 Hz) is close to 0.5Fo, and the center frequency of IMF8 is close to rotational frequency Fr. Through parameter optimization, the IMFs obtained by TVF-EMD have physical meanings, and the signals of main frequency bands are separated.

**Figure 18:**
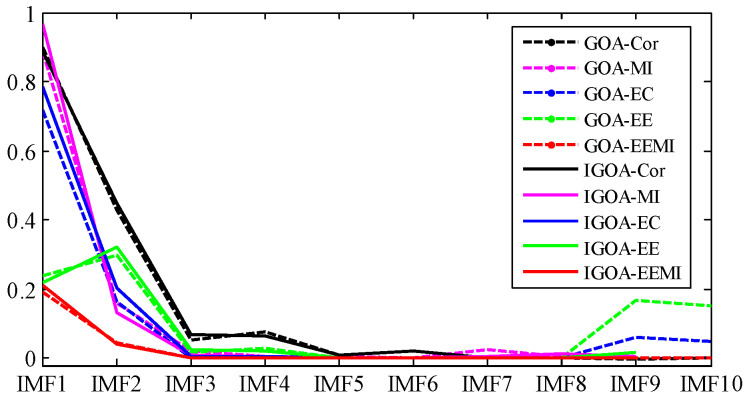
Decomposition results evaluation of outer ring slight damage signal.

Through the TVF-EMD method optimized by GOA, the signal is decomposed into 10 IMFs. The evaluation indexes of the decomposition results are shown in Figure 18 and Table 10. The EC and *EE* indexes of IMF9 and IMF10 obtained by GOA have increased, and their center frequencies are both 5.85 Hz, which indicates that over-decomposition and mode aliasing occurred during the decomposition process. Compared with the indexes obtained by GOA, the indexes of IMF1~IMF9 obtained by IGOA are larger, which indicates that the decomposition performance of IGOA-TVF-EMD is better than that of GOA-TVF-EMD. It can be seen from Table 10 that the difference in RMSE obtained by the two methods is very small, while the absolute value of the ELR index obtained by IGOA is smaller than that of GOA, which indicates that IGOA reduces the decomposition error and energy leakage.

**Figure 19:**
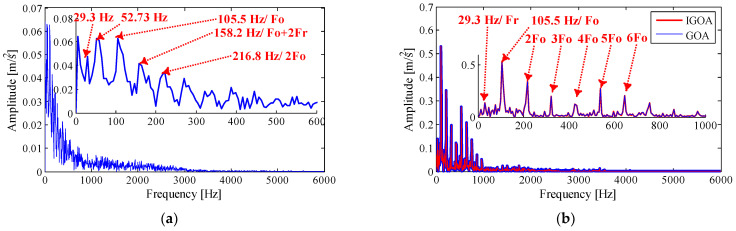
The envelope spectrum of the sensitive IMF obtained by (**a**) TVF-EMD with assigned parameters, and (**b**) TVF-EMD based on IGOA and GOA.

Next, the sensitive IMF is selected according to the kurtosis and its mean threshold, and the demodulation result of the sensitive IMF is shown in Figure 19. The envelope demodulation obtained by the TVF-EMD method with the above specified parameters (ξ=0.2,n=6) is shown in Figure 19a. Although the low-frequency signal is not decomposed successfully, the outer ring characteristic frequency Fo can still be extracted, indicating that the sensitive IMF selection method based on the kurtosis mean threshold is effective. However, the high-order harmonics of Fo are submerged by many interfering frequencies, and satisfactory feature extraction results are not achieved. As shown in Figure 19b, the envelope spectra obtained by GOA and IGOA almost coincide, and the high-order harmonics of Fo can be clearly observed. The results show that the proposed method has good decomposition performance and feature extraction performance.

#### 4.3.2. Analysis of Rolling Element Vibration Signal

**Figure 20:**
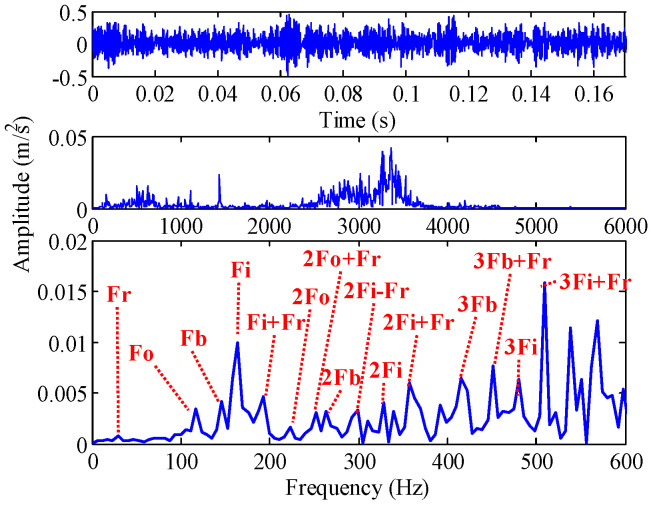
Time-domain and frequency-domain waveform of rolling element slight damage signal.

The time-domain and frequency-domain waveforms of the rolling element vibration signal are shown in Figure 20. The rolling element frequency Fb is submerged by high frequency noise, so we analyze the rolling element refinement spectrum in the range of [0 Hz, 600 Hz]. The spectral peaks of the inner frequency Fi and the modulation frequency 3Fi + Fr are the largest. Next, the rolling element frequencies Fb, 3Fb and the modulation frequency 3Fb + Fr can be observed. In addition, the outer ring frequency Fo and the modulation frequency 2Fo + Fr can also be observed. According to the refined spectrum, the fault state is difficult to identify accurately. This is because the second harmonic of the roller spin frequency is included in the spectrum when the rolling element fails [44]. The roller spin frequency is the result of a faulty roller hitting the inner or outer ring, usually the roller rotates once to produce two shocks. Therefore, the roller failure frequency is easily submerged by the inner ring frequency, outer ring frequency and other components.

**Figure 21:**
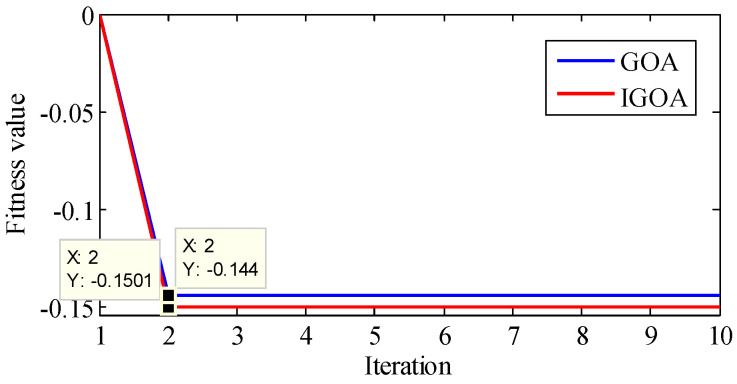
The fitness curve obtained by IGOA and GOA during the decomposition of the rolling element signal.

**Figure 22:**
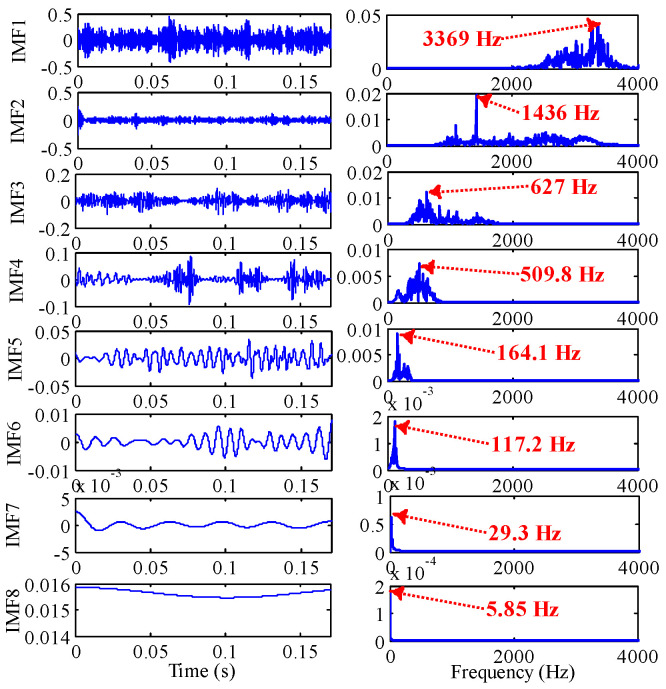
The IMFs of rolling element obtained by the optimized TVF-EMD based on IGOA.

TVF-EMD is optimized by IGOA and GOA, and the convergence curve is shown in Figure 21. The best fitness obtained by GOA is −0.144, and the best parameters are ξ=0.265544, n=16. The best fitness obtained by IGOA is −0.1501, and the best parameters are ξ=0.223517,n=11. It shows that IGOA has better optimization performance. The rolling element signal is decomposed by TVF-EMD optimized by IGOA, and the results are shown in Figure 22. IMF1 and IMF2 are high-frequency noise, IMF4 is close to 3Fi + Fr (516.5 Hz) in Figure 20, IMF5 is close to Fi, IMF6 is close to Fo, and IMF7 is close to rotational frequency Fr. The key components in the signal are effectively decomposed, and these components have specific physical meanings. The results show that the proposed method is effective.

**Figure 23:**
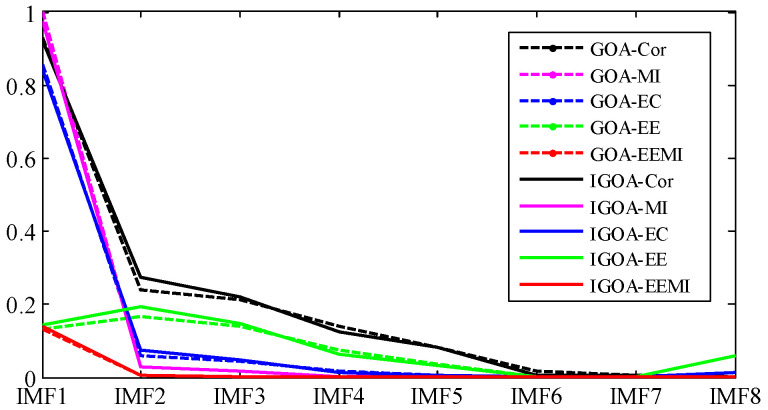
Decomposition results evaluation of rolling element slight damage signal.

Through the TVF-EMD method optimized by GOA, the signal is decomposed into eight IMFs. The evaluation indexes of the decomposition results are shown in Figure 23 and Table 11. Except for IMF4 and IMF6, the Cor, MI, EC, and *EE* indexes of the modes obtained by IGOA all increase, indicating that the decomposition results of TVF-EMD based on IGOA are better. It can be seen from Table 11 that the RMSE index obtained by IGOA is smaller than that of GOA, and the absolute value of the ELR index is smaller. It shows that the proposed method improved the decomposition error and energy leakage. According to the CF index, the approximate value of the outer ring frequency of 117.2 Hz is obtained by the proposed method, while the TVF-EMD based on GOA does not obtain this frequency. However, none of these decomposition methods can obtain the rolling element frequency Fb, because the rolling element frequency Fb is coupled with the inner and outer ring frequencies, and it is very close to the inner ring frequency. Therefore, we select sensitive IMF through kurtosis and its mean threshold, and analyze the reconstructed signal of sensitive IMF by envelope demodulation.

It can be seen from Figure 22 and Table 11 that the Kurt indexes of IMF2, IMF4, IMF6, and IMF7 are relatively large. Because the fault signal is a periodic signal, and Kurt is an index to measure the distribution density of shocks, the IMFs that are larger than the Kurt average are selected as the sensitive IMFs. As shown in Figure 23, if the IMFs with the largest Cor, MI, and EC are selected as the sensitive IMFs, the fault information (such as IMF5, IMF6) will be lost.

**Figure 24:**
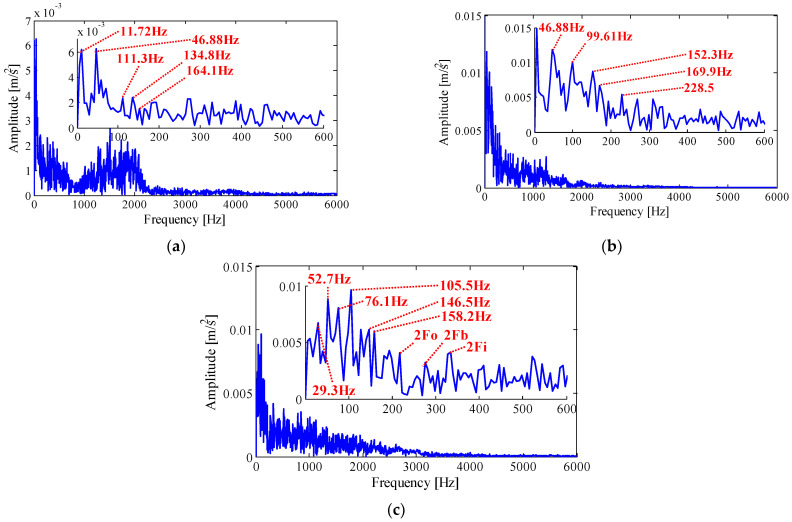
The envelope spectrum of the sensitive IMF obtained by (**a**) TVF-EMD with assigned parameters, and (**b**) TVF-EMD based on GOA; (**c**) TVF-EMD based on IGOA.

The rolling element signal is decomposed by TVF-EMD with specified parameters (ξ=0.1,n=26), and 16 IMFs are obtained by decomposing, and the time-frequency-domain and indexes are no longer listed. The sensitive IMFs obtained by the three methods are analyzed by envelope demodulation, and the results are shown in Figure 24. The envelope spectrum obtained by TVF-EMD with assigned parameters contains interference frequencies at 1000~2000 Hz, indicating that the noise mode is aliased with the effective IMFs. The largest spectral peaks appear at frequencies 11.72 Hz and 46.88 Hz, which are insignificant interference frequencies. The frequencies 111.3 Hz, 134.8 Hz, 164.1 Hz are slightly close to theoretical values Fo (107.36 Hz), Fb (141.16 Hz) and Fi (162.18 Hz), but there is a large deviation between them and the theoretical values, and they are almost submerged in the interference frequency, it is difficult to identify the fault features.

As shown in Figure 24b, TVF-EMD is optimized by GOA, which suppresses the high-frequency noise in the envelope spectrum effectively. The interference frequency of 46.88 Hz is more obvious, and 99.61 Hz, 152.3 Hz, and 169.9 Hz are quite close to theoretical values Fo (107.36 Hz), Fb (141.16 Hz) and Fi (162.18 Hz), but there is a large deviation between them and the theoretical values, and Fr (29.3 Hz) cannot be recognized by this method.

As shown in Figure 24c, TVF-EMD is optimized by IGOA, which suppresses the high-frequency noise in the envelope spectrum effectively. The spectral peaks at frequencies 105.5 Hz and 52.7 Hz are the most obvious, which are close to Fo (107.36 Hz) and 0.5Fo (53.68 Hz). Then, the spectral peaks at frequencies 146.5 Hz and 76.1 Hz are also evident, which are slightly close to Fb (141.16 Hz) and 0.5Fb (70.58 Hz). It shows that the fault features of rolling elements can be extracted. The frequency of 29.3 Hz is close to Fr (29.95) and 158.2 Hz is slightly close to Fi (162.18 Hz). In addition, the spectral peaks of 2Fo, 2Fb, and 2Fi are also obvious compared with other frequency components. The key frequency components in the rolling element signal are almost identified. The results show that the proposed method is effective.

## 5. Conclusions

In order to accurately separate the sub-signals and extract the frequency information in the signal, a parameter-adaptive TVF-EMD feature extraction method based on IGOA is proposed in this paper. First, we introduce a nonlinear decreasing strategy to dynamically adjust the two decreasing coefficients of GOA in this paper. Then, 23 sets of benchmark functions are optimized by IGOA, and the influence of IGOA parameters on the results is discussed. When the coefficient of IGOA is 0.8, its comprehensive optimization performance is the best compared with other methods, and the time complexity is almost unchanged. Then, the *EEMI* index is introduced to comprehensively consider the energy distribution of the modal and the dependence between the IMFs and the original signal, and it is taken as the objective function. Next, TVF-EMD parameters are optimized by IGOA and the optimal parameters matching the input signal are obtained. Finally, sensitive modes with larger kurtosis are analyzed to extract the characteristic frequency of the signal. The proposed method is used to analyze two sets of simulation signals and bearing vibration signals. The results show that: (1) the method can adaptively determine the parameters of TVF-EMD according to the signal features, and it solved the problems of under-decomposition, over-decomposition and mode aliasing of TVF-EMD effectively; (2) the fitness obtained by IGOA is smaller than that obtained by GOA. Compared with GOA and other algorithms, the comprehensive optimization performance of IGOA is stronger, so the proposed IGOA is effective; (3) the *EEMI* index is effective because it considers the energy distribution and correlation of the signal comprehensively, and it is more sensitive to the parameters of TVF-EMD than Cor, Kurt, EC, and *EE*; and (4) the decomposition accuracy of the adaptive TVF-EMD based on IGOA is high, the energy leakage is very small, and meaningful modes can be obtained by decomposition. Therefore, the method proposed in this paper is effective, and the method has potential application value in the field of mechanical fault diagnosis.

## Figures and Tables

**Table 1:** Description of the benchmark functions.

ID	Lower	Upper	Dimension	Type	ID	Lower	Upper	Dimension	Type
F1	−100	100	5	Unimodel	F13	−50	50	5	Multimodal
F2	−10	10	5	Unimodel	F14	−65.536	65.536	2	Multimodal
F3	−100	100	5	Unimodel	F15	−5	5	4	Multimodal
F4	−100	100	5	Unimodel	F16	−5	5	2	Multimodal
F5	−30	30	5	Unimodel	F17	[−5; 0]	[10; 15]	2	Multimodal
F6	−100	100	5	Unimodel	F18	−2	2	2	Multimodal
F7	−1.28	1.28	5	Unimodel	F19	0	1	3	Multimodal
F8	−500	500	5	Multimodal	F20	0	1	6	Multimodal
F9	−5.12	5.12	5	Multimodal	F21	0	10	4	Multimodal
F10	−32	32	5	Multimodal	F22	0	10	4	Multimodal
F11	−600	600	5	Multimodal	F23	0	10	4	Multimodal
F12	−50	50	5	Multimodal	—	—	—	—	—

**Table 2:** Comparison between the IGOAs of different parameters.

F.	IGOA *k1 = k2 = 0.5*				IGOA *k1 = k2 = 1.2*				IGOA *k1 = k2 = 1.5*			
	*Avg.*	*STD*	*Time*	*SR*	*Avg.*	*STD*	*Time*	*SR*	*Avg.*	*STD*	*Time*	*SR*
F1	8.248E−01	3.624E−01	16.21	0	1.068E−11	1.350E−11	15.77	100	4.947E−16	7.003E−16	15.54	100
F2	1.056E+00	1.627E+00	18.72	0	7.758E−01	1.356E+00	15.01	0	7.995E−07	8.764E−10	15.50	100
F3	1.465E+00	8.431E−01	17.38	0	4.338E−04	1.800E−03	16.88	90	4.455E−08	2.235E−07	16.06	100
F4	5.841E−01	1.394E−01	18.96	0	2.316E−06	1.369E−06	18.06	100	1.396E−06	7.471E−06	15.88	100
F5	2.049E+03	3.859E+03	18.60	0	1.574E+03	3.352E+03	17.93	0	1.770E+03	3.272E+03	15.67	0
F6	6.136E−01	2.859E−01	18.03	0	9.769E−12	1.423E−11	20.16	100	6.219E−16	1.008E−15	15.74	100
F7	7.981E−02	1.434E−01	17.88	0	4.300E−02	7.270E−02	15.16	0	4.490E−02	5.330E−02	15.25	0
F8	−1.66E+03	1.893E+02	17.90	100	−1.58E+03	2.036E+02	15.00	100	−1.44E+03	1.983E+02	15.63	100
F9	8.235E+00	5.621E+00	19.49	0	8.253E+00	5.781E+00	15.30	0	6.055E+00	3.893E+00	15.69	0
F10	1.621E+00	6.062E−01	15.77	0	8.167E−01	8.885E−01	17.25	20	7.253E−01	8.899E−01	15.73	23.3
F11	6.152E−01	1.902E−01	18.01	0	2.115E−01	1.224E−01	15.38	0	2.108E−01	1.139 E−01	17.70	0
F12	3.788E−01	4.061E−01	16.59	0	5.490E−02	1.340E−01	17.28	46.6	6.980E−02	1.691E−01	21.36	26.6
F13	5.490E−02	2.290E−02	16.18	0	1.900E−03	4.800E−03	15.88	66.6	3.000E−03	4.900E−03	18.13	60
F14	9.980E−01	2.934E−06	6.10	0	1.262E+00	6.348E−01	5.87	0	1.659E+00	8.767E−01	6.19	0
F15	7.400E−03	1.310E−02	10.83	0	7.200E−03	1.350E−02	10.66	0	8.100E−03	8.900E−03	12.42	0
F16	−1.03E+00	9.892E−06	5.27	100	−1.03E+00	4.554E−16	6.01	100	−1.03E+00	4.701E−16	6.55	100
F17	3.980E−01	2.373E−04	6.39	0	4.043E−01	2.440E−02	5.95	0	4.107E−01	3.320E−02	6.34	0
F18	3.0000	2.434E−05	6.64	0	3.0000	8.137E−15	6.57	0	3.0000	5.405E−15	6.51	0
F19	−3.8626	1.000E−03	11.62	100	−3.8614	1.419E−01	11.41	100	−3.8615	3.100E−03	11.79	100
F20	−3.2703	7.130E−02	20.61	100	−3.2849	6.03E−02	17.47	100	−3.2695	6.58E−02	19.79	100
F21	−6.6352	3.2622	11.77	100	−5.7289	3.0406	11.62	100	−5.7311	3.5128	11.76	100
F22	−6.8842	3.8590	11.94	100	−6.9900	3.1897	11.32	100	−6.8191	3.7311	11.28	100
F23	−7.1422	3.7591	12.34	100	−7.5938	3.7429	11.46	100	−5.7662	3.9768	14.52	100

**Table 3:** Comparison between IGOA (k1=k2=0.8 ) and original GOA.

F.	IGOA *k1 = k2 = 0.8*						GOA					
	*Avg.*	*Best.*	*Worst.*	*STD.*	*Time.*	*SR.*	*Avg.*	*Best.*	*Worst.*	*STD.*	*Time.*	*SR.*
F1	7.71E−05	1.05E−07	5.68E−04	1.58E−04	16.0	80	1.47E−04	1.86E−07	1.70E−03	4.33E−04	15.4	76.6
F2	6.00E−04	4.68E−04	1.89E−02	5.80E−02	15.3	0	8.19E−01	2.80E−03	5.23E+00	1.42E+00	15.8	0
F3	3.50E−03	1.79E−06	1.70E−02	4.10E−03	16.2	40	2.50E+00	4.49E−02	6.02E+00	2.02E+00	15.2	30
F4	1.90E−03	3.55E−04	4.43E−02	1.00E−02	16.4	0	3.70E−03	3.55E−04	2.34E−02	6.10E−02	15.4	0
F5	8.62E+02	9.00E−03	9.31E+03	1.88E+03	16.5	0	7.51E+03	1.97E+00	8.97E+04	2.31E+04	15.5	0
F6	1.22E−04	1.93E−07	1.60E−03	1.10E−03	18.0	80	4.15E−05	3.32E−07	8.30E−04	1.53E−04	15.2	93.3
F7	3.60E−02	7.88E−04	3.35E−01	7.29E−02	16.3	0	7.43E−02	8.90E−04	5.12E−01	1.31E−01	15.1	0
F8	−1.65E+03	−2.0E+03	−1.28E+03	2.03E+02	16.9	100	−1.50E+03	−2.0E+03	−1.14E+03	2.40E+02	15.3	100
F9	6.69E+00	1.45E+00	2.00E+01	4.16E+00	16.0	0	7.28E+00	1.14E+00	1.89E+01	4.77E+00	15.1	0
F10	6.30E−01	1.30E−03	2.40E+00	8.63E−01	16.3	0	9.31E−01	3.64E−04	3.57E+00	9.70E−01	15.1	0
F11	1.57E−01	5.53E−02	4.61E−01	1.11E−01	18.5	0	2.41E−01	4.67E−02	4.63E−01	1.26E−01	15.6	0
F12	6.82E−03	2.29E−07	1.22E−01	2.56E−01	16.9	40	1.41E−01	8.31E−07	1.02E+00	2.63E−01	16.0	20
F13	3.33E−03	1.86E−07	1.52E−02	5.30E−03	17.9	40	5.10E−03	3.68E−06	2.13E−02	6.70E−03	16.0	36.6
F14	1.09E+00	9.98E−01	3.96E+00	5.42E−01	6.5	0	1.55E+00	9.98E−01	6.90E+00	1.24E+00	6.1	0
F15	1.12E−02	3.37E−04	5.92E−02	1.31E−02	6.2	0	1.30E−02	4.28E−04	6.52E−02	1.36E−02	10.6	0
F16	−1.03E+0	−1.03E+0	−1.031E+0	1.32E−11	6.2	100	−1.0E+00	−1.0E+0	−1.03E+00	5.01E−11	5.4	100
F17	3.97E−01	3.97E−01	3.97E−01	7.05E−10	5.9	0	4.04E−01	3.97E−01	4.94E−01	2.44E−02	5.7	0
F18	3.0000	3.0000	3.0000	1.22E−09	5.8	0	3.0000	3.0000	3.0000	5.43E−10	5.4	0
F19	−3.8627	−3.8628	−3.8623	1.37E−04	11.1	100	−3.8614	−3.8628	−3.8229	7.30E−03	10.8	100
F20	−3.2698	−3.3220	−3.1507	6.97E−02	17.7	100	−3.2691	−3.3220	−3.1743	6.05E−02	16.1	100
F21	−6.0565	−1.01E+01	−2.6305	3.2166	14.6	100	−5.4781	−1.01E+01	−2.6305	3.2608	10.8	100
F22	−8.2806	−1.05E+01	−1.8595	3.5643	12.7	100	−7.2992	−1.04E+01	−2.7519	3.6401	10.9	100
F23	−8.2545	−1.05E+01	−2.4217	3.3801	11.6	100	−8.1794	−1.05E+01	−2.4217	3.4411	10.1	100

**Table 4:** The effects of the Maxiter on optimization results.

F.	Maxiter = 100				Maxiter = 200				Maxiter = 300			
	IGOA		GOA		IGOA		GOA		IGOA		GOA	
	*Avg.*	*SR.*	*Avg.*	*SR.*	*Avg.*	*SR.*	*Avg.*	*SR.*	*Avg.*	*SR.*	*Avg.*	*SR.*
F1	1.700E−03	0	2.600E−03	0	9.000E−07	100	3.885E−07	100	3.524E−07	100	1.042E−087	100
F2	1.142E+00	0	2.547E+00	0	7.715E−01	0	1.916E+00	0	9.618E−01	0	1.913E+00	0
F3	1.180E−02	0	1.450E−02	0	5.978E−04	46.67	2.000E−03	0	1.590E−05	100	9.841E−05	96.67
F4	2.320E−02	0	4.490E−02	0	6.343E−04	0	6.681E−04	0	2.908E−04	0	2.659E−04	0
F5	8.360E+02	0	2.141E+03	0	8.757E+02	0	1.109E+03	0	3.757E+02	0	1.462E+03	0
F6	2.700E−03	20	2.100E−03	20	7.975E−07	100	8.021E−07	100	2.574E−07	100	1.806E−07	100
F7	3.940E−02	0	1.654E−01	0	2.180E−02	0	7.180E−02	0	3.700E−02	0	8.920E−02	0
F8	−1.59E+003	100	−1.50E+03	100	−1.67E+03	100	−1.61E+03	100	−1.59E+03	100	−1.53E+03	100
F9	6.783E+00	0	10.22E+00	0	5.999E+00	0	10.8E+00	0	5.923E+00	0	6.379E+00	0
F10	6.841E−01	0	1.068E+00	0	7.283E−01	0	1.06E+00	0	1.701E−01	0	6.653E−01	0
F11	2.308E−01	0	1.80E−01	0	1.788E−011	0	1.78E−01	0	1.914E−01	0	1.752E−01	0
F12	1.340E−01	0	1.951E−01	0	1.360E−02	16.67	2.800E−02	0	5.200E−03	56.67	1.740E−02	43.33
F13	1.600E−03	13.44	4.700E−03	0	3.100E−03	43.33	3.500E−03	36.67	2.90E−03	63.33	4.200E−03	40
F14	1.295E+00	0	1.29E+00	0	9.980E−01	0	9.980E−01	0	9.980E−01	0	9.980E−01	0
F15	2.000E−03	0	2.300E−03	0	2.800E−03	0	6.200E−03	0	2.900E−03	0	5.000E−03	0
*Avg.T*	10.96s		11.03s		21.29s		21.01s		32.02s		31.68s	

**Table 5:** The effects of the population size on optimization results.

F.	Population = 30				Population = 100				Population = 200			
	IGOA		GOA		IGOA		GOA		IGOA		GOA	
	*Avg.*	*SR.*	*Avg.*	*SR.*	*Avg.*	*SR.*	*Avg.*	*SR.*	*Avg.*	*SR.*	*Avg.*	*SR.*
F1	6.641E−05	80	9.246E−05	80	1.755E−04	50	2.710E−04	70	3.000E−03	0	1.600E−03	0
F2	1.021E+00	0	1.045E+00	0	8.316E−01	0	1.604E+00	0	5.372E−01	0	1.941E+00	0
F3	9.100E−03	0	2.030E−02	0	3.000E−03	0	5.900E−03	0	4.300E−03	0	4.300E−03	0
F4	3.100E−03	0	3.600E−03	0	1.040E−02	0	7.200E−03	0	3.840E−02	0	2.820E−02	0
F5	1.059E+03	0	9.489E+02	0	1.289E+02	0	4.123E+02	0	1.694E+02	0	6.788E+01	0
F6	1.064E−04	90	1.052E−04	90	8.011E−05	90	3.240E−04	50	1.300E−03	0	3.300E−03	0
F7	3.180E−02	0	5.750E−02	0	2.460E−02	0	2.860E−02	0	1.100E−03	0	5.600E−03	0
F8	−1.43E+03	100	−1.43E+03	100	−1.70E+03	100	−1.66E+03	0	−1.64E+03	100	−1.50E+03	100
F9	1.211E+01	0	1.452E+01	0	4.323E+00	0	7.984E+00	0	4.599E+00	0	4.705E+00	0
F10	7.577E−01	0	1.431E+00	0	4.363E−01	0	4.799E−01	0	5.496E−01	0	9.241E−01	0
F11	2.665E−01	0	3.428E−01	0	1.683E−01	0	2.279E−01	0	1.886E−01	0	1.936E−01	0
F12	2.300E−02	20	6.650E−02	10	7.908E−04	40	1.900E−03	20	3.100E−04	33.33	1.600E−03	30
F13	4.500E−03	53.33	5.000E−03	50	1.100E−03	33.33	5.900E−03	30	2.200E−03	20	6.200E−03	0
F14	1.295E+00	0	1.395E+00	0	9.980E−01	0	9.980E−01	0	1.097E+00	0	1.295E+00	0
F15	1.540E−02	0	1.550E−02	0	6.800E−03	0	6.900E−03	0	0.0054E−03	0	3.300E−03	0
*Avg.T*	6.03s		6.20s		61.79s		60.66s		254.41s		253.91s	

**Table 6:** Decomposition results evaluation of simulated signal x(t) under different parameters.

Index	Assigned Value (ξ=0.1,n=20)				GOA (ξ=0.174669 ,n=23)			IGOA (ξ=0.145251 ,n=23)		
	IMF1	IMF2	IMF3	IMF4	IMF1	IMF2	IMF3	IMF1	IMF2	IMF3
COR	0.25964	0.43251	0.39650	0.77542	0.25966	0.43225	0.86390	0.25966	0.43238	0.86391
MI	0.80834	1.02542	0.28548	0.88980	0.69371	1.06834	2.37064	0.74940	1.13667	2.61562
Kurt	2.91748	2.92008	1.68794	2.06884	2.91778	2.91497	2.91509	2.91797	2.91691	2.91567
EC	0.06798	0.18881	0.14698	0.59621	0.06715	0.18655	0.74628	0.06716	0.18656	0.74627
EE	0.18277	0.31475	0.28183	0.30833	0.18137	0.31323	0.21839	0.18138	0.31323	0.21840
EEMI	0.14774	0.32275	0.08045	0.27435	0.12581	0.33463	0.51773	0.13593	0.35604	0.57127
CF	200 Hz	100.3 Hz	54.93 Hz	49.8 Hz	200 Hz	100.3 Hz	49.8 Hz	200 Hz	100.3 Hz	49.8 Hz
RMSE	6.63254372362072E−17				6.10663958406721E−17			5.52830577450765E−17		
ELR	1.26756751554804E−2				6.11789560325569E−4			6.07251242923508E−4		

**Table 7:** Decomposition results evaluation of simulated signal y(t) under different parameters.

Index	GOA (ξ=0.117211 ,n=17)					IGOA (ξ=0.107442 n=27)				
	IMF1	IMF2	IMF3	IMF4	IMF5	IMF1	IMF2	IMF3	IMF4	IMF5
Cor	0.05406	0.04420	0.81011	0.53668	0.22806	0.05491	0.04438	0.81015	0.53678	0.22820
MI	1.050E−4	5.276E−4	0.84841	1.19674	0.53840	2.5624E−3	1.484E−3	0.84715	1.21227	0.55248
Kurt	3.14098	3.76649	1.52020	1.50596	1.52899	3.11645	3.60330	1.52116	1.50587	1.52822
EC	0.00290	0.00188	0.65602	0.28751	0.05166	0.00299	0.00190	0.65601	0.28749	0.05158
EE	0.01698	0.01180	0.27655	0.35838	0.15308	0.01740	0.01195	0.27655	0.35838	0.15292
EEMI	1.784E−6	6.228E−6	0.23463	0.42889	0.08241	4.460E−5	1.774E−5	0.23428	0.43445	0.08448
CF	472.2 Hz	310.1 Hz	75.2 Hz	24.9 Hz	15.14 Hz	472.2 Hz	310.1 Hz	75.2 Hz	24.9 Hz	15.14 Hz
RMSE	1.63490379207628E−16					1.60185216006952E−16				
ELR	1.40291463966830 E−3					1.38564962092271E−3				

**Table 8:** The bearing structure factor of SKF 6205.

Bearing Model	Rolling Element Diameter	Number of Rolling Element	Bearing Pitch Diameter	Contact Angle
SKF6205	7.938 mm	9	39 mm	0

**Table 9:** Fault characteristic frequency (unit: Hz).

Fr	Fi	Fo	Fb
29.95	162.1852	107.3648	141.1693

**Table 10:** Decomposition results evaluation of outer ring slight damage signal under different parameters.

Index	GOA (ξ=0.28026 ,n=7)									
	IMF1	IMF2	IMF3	IMF4	IMF5	IMF6	IMF7	IMF8	IMF9	IMF10
Kurt	7.986	16.944	6.350	2.6619	2.8834	2.1094	4.0275	3.4262	6.3318	6.3307
CF	3445 Hz	2801 Hz	1400 Hz	539.1 Hz	269.5 Hz	164.1 Hz	58.59 Hz	29.3 Hz	5.85 Hz	5.85 Hz
RMSE	8.73523111467058e−17									
ELR	−0.122195409491517									
Index	IGOA (ξ=0.238489,n=15)									
	IMF1	IMF2	IMF3	IMF4	IMF5	IMF6	IMF7	IMF8	IMF9	—
Kurt	7.478	12.507	6.240	3.6839	2.796	2.220	4.640	2.1756	5.5451	—
CF	3445 Hz	2906 Hz	1400 Hz	539.1 Hz	269.5 Hz	164.1 Hz	58.59 Hz	29.3 Hz	23.44 Hz	—
RMSE	8.75022651069671e−17									
ELR	−0.000118428965832907									

**Table 11:** Fault characteristic frequency (unit: Hz).

Index	GOA (ξ=0.265544 ,n=16)							
	IMF1	IMF2	IMF3	IMF4	IMF5	IMF6	IMF7	IMF8
Kurt	3.0203	10.443	3.3474	3.8239	2.5435	9.8020	8.7031	1.8026
CF	3369 Hz	1436 Hz	627 Hz	509.8 Hz	164.1 Hz	70.31 Hz	29.3 Hz	5.85 Hz
RMSE	1.67122144577936e−17							
ELR	−0.000896154006018536							
Index	IGOA (ξ=0.223517,n=11)							
	IMF1	IMF2	IMF3	IMF4	IMF5	IMF6	*IMF7*	*IMF8*
Kurt	2.9835	9.3753	3.4777	6.3255	2.8033	3.5137	6.4838	1.7957
CF	3369 Hz	1436 Hz	627 Hz	509.8 Hz	164.1 Hz	117.2 Hz	29.3 Hz	5.85 Hz
RMSE	1.67116896663240e−17							
ELR	−0.00042497562128863							

## Data Availability

The data used to support the findings of this study are available from the corresponding author upon request.
